# Can the Increase of Insanity Be Prevented?

**Published:** 1912-07

**Authors:** Wilmer L. Allison

**Affiliations:** Fort Worth, Texas


For Texas Medical Journal.

### Can the Increase of Insanity be Prevented?

BY WILMER L. ALLISON, M. D., FORT WORTH, TEXAS.

To the writer there is no better sign of the progress of the times than that shown by the closer association of the physician and the public.

Necessarily our entire lives are closely associated with the physician, but in the past there has been lacking that close association which the recent organization of the profession in this country is bringing about.

The physician, by virtue of his training and the nature of his profession, stands rightly in the light of an adviser, not only to that part of humanity that is already suffering, but also to those who are enjoying the blessings of good health, that they may be the better advised how to remain well and strong and happy.

The true field of the physician is rather to prevent disease and suffering than to alleviate that suffering when it comes.

In the dawn of our existence, when we cross the threshold of life, in all our ignorance and helplessness, the physician is with us to guide and teach us. As the sun of our lives reaches its meridian heights, he is ready there to make our happiness brighter by pushing us out of the shadows of disease and suffering and holding us in the bright sunlight of truth and knowledge of our own selves and the laws which govern our bodies. And at the sunset of life he is there ready to steady our footsteps down the declining slope which leads to the grave.

The public is gradually but surely coming to know that the profession of medicine stands ever ready and willing to help ward off all sickness and suffering, though this public often accuses us of trying to further our financial gain when we ask for protective laws and measures to safeguard the public health, while these laws, if passed and enforced, would lessen our opportunities in that particular direction, still with the rapid increase of our population and civilization, new diseases and new problems are constantly arising which must engage our attention, so that the doctor's work is never done.

Now that we are constantly associated with the physician who is ready at all times to do all things in his power to make our paths easy and free from disease and suffering, why not consult him in vital matters pertaining to our health and the health of the community?

After showing you that we are qualified to speak, we will try to show you that the question, "Can the increase of insanity be prevented?" can be answered in the affirmative and why, by showing you why mental disease is increasing; then the remedy will suggest itself.

That mental disease is increasing, and at an alarming rate, there is no question. In the past few years our public institutions have been doubled in size, and yet during the past ten years every institution in our State has been overcrowded, and the jails have been more or less taxed over the entire State. The writer has frequently heard county judges say that they had from ten to twenty-four insane in their jails, and no room for them in the asylums. Without discussing the absolutely inhuman fact that these mentally sick are even sent to the jails and compelled to stay there waiting for room, suffice it to say that they sometimes have to remain there for months at a time, until they often lose what chance they had of getting well. And all this because of such an increase in mental disease that our Legislatures have failed to realize the situation and provide sufficient room.

It may occur to you that this increase is because the increase of our population is so rapid, and that is partly true, but the fact remains that the per cent increase of mental disease has far exceeded the per cent increase of the population. Fifty years ago in this State there was one insane person to every 12,800 population. The normal proportion now is one to every 300. No wonder that taxation is becoming a problem, for these unfortunates have to be cared for by those who are more fortunate.

One of our prominent authorities makes the statement that the chief factors in the causation of insanity may be summed up in two words—heredity and strain. Heredity is responsible for the instability of the nervous system. In the present time of quick marriages and quick divorces, we know that too little attention is paid to the selection man makes in his choice of a wife or woman makes in the choice of a husband.

How careful we are, even the most ignorant farmer, to properly mate our stock, even the hogs which are killed as soon as large enough. Yet how careless we are in our own human selections. We see about us thousands of evidences of the degeneration of the race. These physical signs of degeneration preponderate in the insane, and is it not reasonable to expect mental degeneration where so much physical degeneration can be seen?

By far too many people marry who are unfitted for the cares and trials which married life brings. Too many enter this most holy of alliances wholly ignorant of its meanings and responsibilities, and unfitted from every standpoint to bring into the world more individuals whom they can not possibly train properly. It is a true saying, that if you will bring up a child in the way that it should go it will not depart from it. And yet how many parents do we see who do not and can not possibly bring up their children in the way they should go, because they themselves are ignorant and unfit. There is no longer any question whether ignorance is a crime. Many authorities have demonstrated the close relationship between ignorance and insanity and crime.

If you will investigate the cases of mental disease in our State institutions you will be surprised to learn how few have ever been well educated and how many are lacking even the rudiments of an education. Then how much more ignorant must they be of their own bodies and the laws of nature. The parent must know something of his own body and the laws which govern it to properly educate his child to select for himself the proper mate.

No doubt some of you will say that love is all in marriage and that it is wrong to select a mate for his or her good qualities and that love should decide, but it is my belief that the man who marries, for love, an undesirable mate would not find himself so in love with this same mate were he better educated to see the signs of degeneration and those qualities in her which as surely means that her children must necessarily be weaklings as it means that the offspring of a poorly developed shabby work horse can not possibly make good race horses even though the sire may be of the best breed.

There is much in heredity, and it will be appreciated and studied only when we pay more attention to these matters in education.

When we say that much of the cause of mental derangement in this or that patient is due to heredity, we do not mean that the father or mother or some other relative has been insane, though this is frequently the case, but we mean that the patient has been born with unstable nervous organization. He has not inherited that stable and well-balanced central nervous system he should have.

Nature has so willed it that we have some natural powers of selection in that we fall in love with those whose inherent tendencies are more or less different from our own, and our instabilities are often corrected and counteracted in this way, but in the haste of our uncontrolled and improperly educated passions we usually pay too little heed to nature's warnings.

Those who are in bad health or suffering from some chronic disease such as tuberculosis, or those who are mentally weak or suffering or have suffered with insanity or some hereditary nervous affection should not be allowed to bring children in the world, the majority of whom will be degenerates or possibly weak-minded, to say nothing of the fact that such a parent would be unfit for their care and training. There are numerous cases in history of insane or criminal persons from whom have come many descendants who have been a burden on society and the State.

Allow me to mention one such case—that of Max Jukes—the drunkard and degenerate who was born in New York in 1720. Of his descendants, 1206 have been identified as inmates of penal and charitable institutions; 310 were inmates of poorhouses on the average of over seven years each; 440 viciously diseased; 56 were notorious public prostitutes; 7 were convicted murderers; 60 were habitual thieves and 134 were habitual criminals. They cost society over a billion and a quarter dollars. Think of it! you who live in this day and time when the dollar means more than human lives or human suffering. Why from a financial standpoint alone it would have been better had Max Jukes never been born. Then in contrast consider the family of which Robert E. Lee was an illustrious member. If I remember right, something over three thousand members have been traced and not one of them has ever been an inmate of an asylum, jail or poorhouse. Blue blood means good heredity, good environments, and good training.

But you ask what can we do to prevent it? As has been mentioned, education of yourselves and your children will do much to relieve the situation, but there are many in the present generation whom education will not reach. And let me say here that by education we do not mean simply an education along literary lines but that broad education which will teach the child about its own body functions and its proper relations to its fellow beings.

Society has the right to demand that it be protected, and our State government has the right to protect it. There is in force, in at least one State, and possibly in others, a law which is intended to check the procreation of undesirable and degenerate offspring. This law requires that a slight operation be done to certain unfit individuals which will effectually stop reproduction without interfering with the sexual power of the man or woman. Now since certain individuals should not be allowed to procreate, the government should compel these operations on the insane and mentally unfit. What would be the result? The class of degenerates and mentally unfit would rapidly disappear, society would be relieved of much of its burden of caring for them, and every man, woman and child within the borders of our State would feel the happy results. The remedy may at first seem too severe, but we have shown how desperate the situation is, and after careful thought you will see that the remedy is not severe at all.

But let us speak of the child itself. Since a great majority of the insane have back of them some hereditary influence, it is our duty to properly protect and safeguard the growing child, especially those who are predisposed. There is no question that the proper education and training of the predisposed child will do much toward warding off approaching trouble. Its training must be directed from every standpoint to insure a strong, healthy, robust adult. And who better than the physician can give us the proper advice in this direction?

It is to be regretted that the family physician is not more often consulted in this matter. It is also to be regretted that the family physician does not exist now as he did in former times. We mean the physician that has been associated with your family for years, who was present at your birth and the birth of your children, and who is frequently consulted on all matters pertaining to the proper bring-up of your children. The family physician, by virtue of his long association with your family, is better acquainted with your idiosyncrasies and weaknesses and predicpositions.

Infections in early life have much influence. During this time the child always should be under the care of a physician and every precaution taken to avoid dangerous sequelae. Typhoid fever, as well as other infectious diseases, too often leaves behind a crippled mind and nervous system, and often because the doctor's orders were laxly obeyed. But in this day of patent medicines the doctor is often not consulted at all or when it is too late for his services to do any good.

The habit of the child eating piecemeal and between meals and all kinds of indigestible substances has often been the starting point of epilepsy or severe headache. Constantly threatening the child with the policeman and the bogy man, scolding harshly or allowing the child to have its own way because it frets and cries, all have their deleterious effect on the tender nervous system. How many children in the first few months of their lives are kept in a constant state of indigestion and bad health because they are nursed at all hours of the night or day or whenever they cry for it. They are too many to be counted, and yet during the time when they should have the best start they are dwarfed and held back both mentally and physically. The over-petted and spoiled child is a menace to its own future.

Improper associations during the tender years, from which are learned bad habits and sensational or exciting stories from which come an utter disregard for the rights of others and cruelty to the lower animals and a lack of respect for the decent things of life that are worth while. The moving picture show with its unnatural stimulus to the tender imagination is a new factor that also needs to be dealt with.

Vulgar or coarse knowledge of the sex relations, which should be properly explained to the child in all its holy and sacred relationship, often leads to bad and damaging habits, all of which have their deleterious influence on the future mentality.

Did you ever notice an individual whose hardened facial expression and appearance led you to remark, "Poor fellow, he is not to blame; he did not have the proper training and associations, and you can expect nothing good from him"? And did you ever look into the kind and pleasant face of some man or woman and see the cheer and pleasure there, and realize that their training was good and their thoughts and associations pure?

Then, too, any pathological condition found in the child should be corrected. Phimosis in both boys and girls should be corrected, for it is often the cause of much nervous reflex disturbance and bad habits.

Any error of the eyes should be looked after, for they are often responsible for the backward mental development of the child. Adenoids and enlarged tonsils with their accompanying result, mouth-breathing, have stunted many a mind and body that should be looked after early.

Sometimes the mistake is made of crowding a child in its books, music, and that often at a time when he is not strong enough mentally and physically to do the work. It is not always the amount of education but the proper direction of that education that is needed. It is better to have a strong, healthy laboring child than a highly specialized neurotic.

In the beginning, we spoke of strain as a cause of the increase of insanity. In the good old days several decades ago there were but few nervous breakdowns. People took things easier. Their life was less strenuous than now. But the present age is a mercenary one. To grasp and to hold is our motto. It is not how well we live but how fast.

The care which accompanies business worries and failures, domestic troubles and love affairs, the habitual use of damaging drugs such as morphine and cocaine and whisky, all with their accompanying lack of sleep and rest, is often responsible for digestive troubles, insomnia and a condition of nervous exhaustion. Blood changes are apt to follow such anemia and a cachectic state, the individual is easily fatigued and there often follows a real mental breakdown. Given a predisposition to nervous breakdown from a faulty heredity basis and training and the strain we keep ourselves constantly under at the present will do the rest. Strong indeed is the man who does not break down.

Alcohol is said to be the direct cause of from 18 per cent to 20 per cent of the insanities in males. In women it is much less important as a cause, though it is a sad fact that even they succumb to its influence occasionally.

As an indirect cause through the untold suffering and misery it brings to all related to its user we have no knowledge of knowing. But considering the poverty and miserable conditions it brings about, as well as its bad hereditary influence, we have every reason to believe that it is much more frequently a cause of mental disease than above noted.

The moral causes of insanity, such as grief, worry, care, domestic troubles, etc., mentioned above under the head of strain, are said to be responsible for 24 per cent of all cases. Many of these troubles can be laid at the door of improper training and hasty marriages.

And last, but not least, the great black plague that is now menacing our country is in a large measure responsible for many of the insane. The young man who sows his wild oats must reap the sad result when he comes to later years. Many patients suffering with specific troubles are not properly warned by their physicians that in order to get well their treatment must extend over a period of two or three years, and many others, thinking the physician is a grafter because he insists on further treatment after all visible signs of the disease have disappeared, discontinue their treatment, only to develop in a few years some incurable mental and nervous trouble. Such patients should be required to have a physician's certificate stating that they are well before being allowed to marry. Then the wives and children would not have to suffer for the wrongs of the father.

A great deal more might be said along these same lines, but we hope that you will have gained something from what has been said, and that you will lend your moral support to any measure that may be suggested for the lessening of mental disease and suffering.

